# Electromagnetic Control System for Capsule Navigation: Novel Concept for Magnetic Capsule Maneuvering and Preliminary Study

**DOI:** 10.1007/s40846-015-0055-2

**Published:** 2015-08-06

**Authors:** Gioia Lucarini, Marco Mura, Gastone Ciuti, Rocco Rizzo, Arianna Menciassi

**Affiliations:** The BioRobotics Institute, Scuola Superiore Sant’Anna, Pisa, Italy; Department of Energy and Systems Engineering, University of Pisa, Pisa, Italy

**Keywords:** Electromagnetic system, Gastrointestinal endoscopy, Magnetic medical devices

## Abstract

The gastrointestinal tract is home of some of the most deadly human diseases. The main problems are related to the difficulty of accessing it for diagnosis or intervention and concomitant patient discomfort. The flexible endoscopy technique has established itself in medical practice due to its high diagnostic accuracy and reliability; however, several technical limitations still remain and the procedure is poorly tolerated by patients. The use of magnetic fields to control and steer endoscopic capsules is increasing in minimally invasive procedures. In fact, magnetic coupling is one of the few physical phenomena capable of transmitting motion beyond a physical barrier, allowing for the compact design of the device itself. In this framework, the authors present the preliminary design and assessment of a magnetic coupling for magnetic endoscopic capsules considering an electromagnetic approach. In particular, a novel toroidal electromagnet is proposed as the control and driving system. The system concept, design, and preliminary results are reported.

**Electronic supplementary material:**

The online version of this article (doi:10.1007/s40846-015-0055-2) contains supplementary material, which is available to authorized users.

## Introduction

The gastrointestinal (GI) tract is home of several of the most deadly human diseases [1]. The survival rate can reach 90 % in cases of early diagnosis, and regular screening of the GI tract is highly recommended for people older than 50 years [2]. Flexible endoscopy has established itself as the standard method due to its diagnostic accuracy and reliability, but several limitations still remain. The physician needs a lot of practice to acquire the necessary skills to manipulate the endoscopic tool, and the movements performed by the endoscope inside the body are frequently painful and traumatic and poorly tolerated by patients. General anaesthesia may be a solution to these discomfort problems, but it is not always possible considering the patient’s age and clinical history and the associated costs. With the introduction of wireless capsule endoscopy (WCE) in 2000, a new screening method with optimal comfort became available [3]. This procedure consists of a small capsule with an embedded camera that, after being swallowed, transmits images to a storage device outside the body. Several companies produce “smart pills”, but there are limitations, mainly related to the unpredictable and uncontrollable locomotion of the capsule [4]. As an intrinsic limitation, WCE does not allow the operator to control navigation. The movement of the capsule is passive and the capsule proceeds by means of visceral peristalsis and gravity. This makes the trajectory of the capsule uncontrollable and unpredictable, so that some portions of the GI internal wall are unlikely to be visualized. Another drawback of WCE is the fact that, if areas of clinical interest are identified, the endoscopist cannot manoeuvre the capsule locally for detailed inspection. All these factors contribute to dramatically limit the diagnostic outcome. Therefore, solving the manoeuvring problem would significantly enhance the accuracy and reliability of endoscopic investigation with an expected improvement of diagnostic efficacy.

Using magnetism for the actuation and localization of endoscopic capsules in the GI tract has become an active area of research and it is a promising approach to solve the problem of control [5, 6]. Two approaches for achieving the magnetic control of a magnetic capsule have been used in recent years: (i) using one or more permanent magnets [7–10] and (ii) using one or more electromagnets (EMGs) [11, 12]. Nevertheless, despite the growing scientific interest in magnetically controllable WCE, no effective system with straightforward clinical applicability has yet been demonstrated. EMGs have the major advantage of delivering controlled fields with no moving parts, and they can be designed in a variety of ways to create spatially uniform magnetic fields and gradients. Permanent magnets, however, can apply clinically relevant forces and torques to a magnetic device, inexpensively and in a compact form-factor without the use of high electrical currents, which are required to drive EMGs. For permanent magnets, the magnetic field can be modulated by translating or rotating one or more external magnets, but this is constrained by the complex geometry of the produced dipole field. The produced field cannot be removed without moving the external magnets very far away from the workspace or shielding the magnet itself [13].

In this framework, the authors present a feasibility study and assessment for the design of a novel electromagnetic system for capsule endoscopes. Although a preliminary approach has previously been presented [14], here the authors report an accurate proof-of-concept of the system together with an accurate experimental evaluation. The primary difference between the proposed EMG system and existing magnetic control platforms [7–10] is the combination of the advantages of electromagnets with the flexibility of robotic manipulation in order to reduce the number of electromagnetic modules and thus the size of the related hardware while maintaining tunable and reliable control of the magnetic force in the operating workspace.

A toroidal EMG with an inner ferrous core is proposed as the WCE control system, which can possibly be manoeuvred by the physician through a manipulator. This particular configuration allows (i) optimizing the EMG dimensions and geometry for high magnetic field generation and, at the same time, (ii) getting the magnetic poles close to each other for more reliable control of the capsule.

## Materials and Methods

### System Overview: Concept of Magnetic Control Platform

**Fig. 1:**
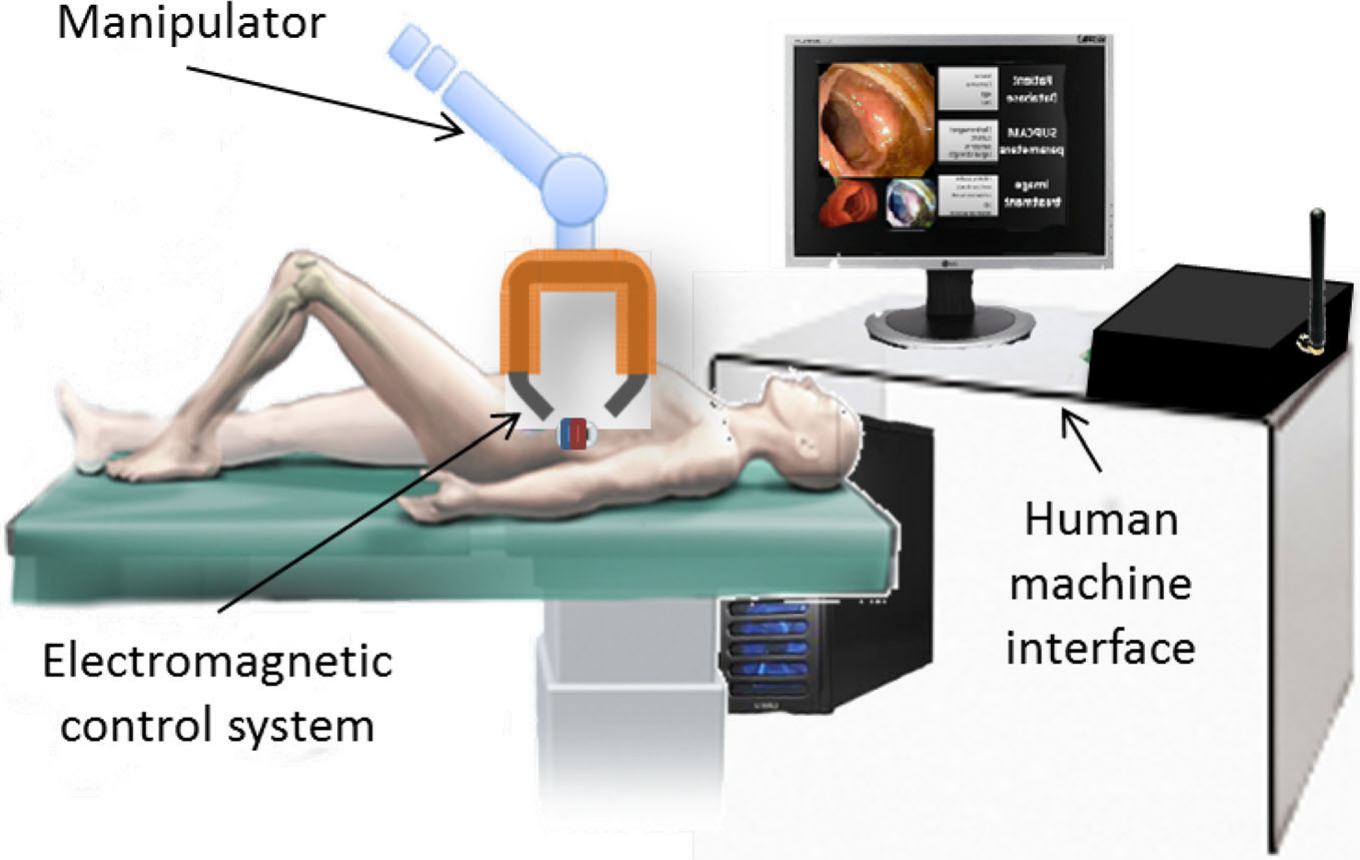
Electromagnetic control system for capsule endoscope navigation. Medical doctor controls endoscopic magnetic capsule inside patient by means of magnetic link with external toroidal EMG, which is supported by a manipulator

The proposed platform is composed of two main modules: (i) a magnetic endoscopic capsule with dimensions proper for navigation and diagnosis throughout the colon (diameter of between 30 and 60 mm [15]), and (ii) an external driving system that includes a toroidal EMG (a schematic representation of the control platform is represented in Fig. 1). Operatively, the physician introduces the endoscopic capsule through the rectum of the patient and insufflates the colon with air for lumen distension. After that, the medical doctor performs the diagnostic procedure by navigating and steering the camera capsule along the colon using the external magnetic manipulator as the driving source.

The toroidal EMG is intended for general use in the magnetic endoscopic capsule procedure and thus only specifications and features significant for its design are here reported. The endoscopic capsule integrates a permanent magnet that can generate a magnetic field that interacts with the external electromagnetic source. Therefore, considering (i) the capsule internal space constraints due to the integration of all components (e.g., battery, electronics boards, telemetry, camera, etc.), (ii) the anatomical constraints of the colonic tissue (i.e., the friction coefficient and diameter of the colon, which can range respectively from 0.1 to 0.7 and from 30 to 60 mm depending on the operation conditions [16], and the distance from the abdominal wall, which can range from 70 to 100 mm [17]), and (iii) the required degrees of freedom (DoFs) for performing a diagnostic analysis (3 DoFs for position and 2 DoFs for orientation), a diametrically magnetized permanent magnet with a volume of 500 × 10^−7^ m^3^ (corresponding to about 5 % of the total capsule volume) was chosen. A neodymium magnet (NdFeB, N52 with a magnetization of 1.48 T) was taken in consideration because neodymium magnets are the strongest type of permanent magnet available. A rough yet conservative prediction of the capsule weight, necessary for the design of the external EMG, may be two times the Given Imaging PillCam^®^ weight (around 4 g), which is considered a golden standard for WCE [18].

The external toroidal EMG, the principal focus of this paper, has to be able to generate suitable forces and torques for three-dimensional (3D) navigation in the presence frictional and deformable GI tract tissues, in a range of distances between the external magnet and the endoscopic device that are related to the abdominal thickness (i.e., 70–100 mm, [17]). Moreover, for fine control of pitch (visualization of upper and lower wall of the colon) and yaw (visualization of right and left wall of the colon), specific magnetic torques have to be properly generated on the capsule. As well as the requirements in forces and torques, constraints of compatibility with the medical application and environment need to be considered. In particular, the electromagnetic system needs to match the normal outpatient setting. Compactness of the external EMG is thus of most importance. Moreover, the electromagnetic system has to be portable and easily controllable by the physician for generating a proper link with the endoscopic device with smooth motion control (potentially assisted by a manipulator for teleoperated or human-cooperative control). In agreement with physicians, the size of the electromagnetic system was fixed to a maximum volume of 200 × 200 × 200 mm^3^.

### Scenario for Magnetic WCE Force and Torque Requirements

**Fig. 2:**
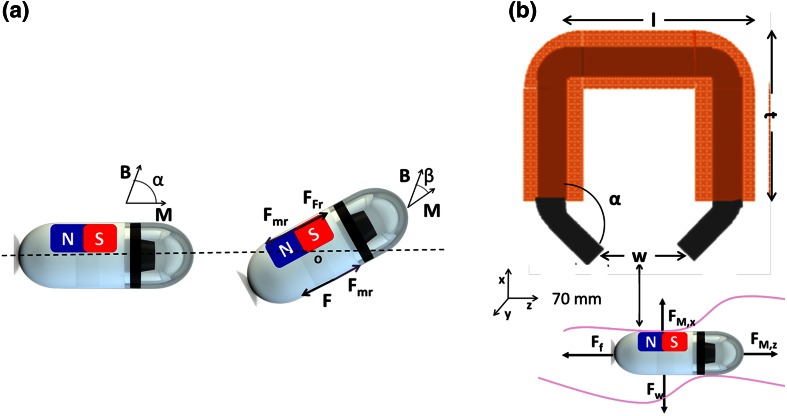
**a** Initial generic orientation of magnetic capsule and field-driven rotation, quantified by angle *β* between final and initial directions of magnetization M; problem is schematized with two equivalent couples of rotational forces *F*_*mr*_ (magnetic couple) and *F*_*fr*_ (frictional couple). **b** Design of EMG parameters to be optimized together with model of forces acting on capsule. *F*_*M*_,_*x*_, *F*_*M,y*_, *F*_*w*_, and *F*_*f*_ are the magnetic attraction force, magnetic dragging force, gravity force, and friction force of the device, respectively. Coils are represented in *copper color*, whereas iron core is *grey*. xyz coordinate system is local reference system related to capsule

A magnetic capsule has to experience both torque and force via interaction with an external magnetic field with flux density *B* (T). The magnetic torque *τ* (Nm) can be expressed as [19]:

1 $$\vec{\tau }_{M} = V\vec{M} \times \vec{B} = V\left[ {\begin{array}{*{20}c} {M_{x} B_{x} } \\ {M_{y} B_{y} } \\ {M_{z} B_{z} } \\ \end{array} } \right]$$

where V (m^3^) and M (A/m) are the volume and the magnetization of the object, respectively. This torque tends to align the magnetization of the magnetic capsule (*M*) to the applied external magnetic field (*B*). With *α*, the initial angle between *M* and *B*, and β, the magnetically induced rotation angle of the device around a fulcrum *O* (Fig. 2a), the modulus *τ*_*M*_ of the magnetic torque is obtained as:

2 $$\vec{\tau }_{M} = VMB\sin \left( {\alpha - \beta } \right)$$

The rotation can be described in terms of an equivalent couple of magnetic rotational forces *F*_*Mr*_. Each rotational force can be considered to be applied to the centroid of the capsule’s half-volume on which it acts. Due to shell homogeneity, each centroid can be assumed to be at the middle point between the fulcrum and the capsule extremity (Fig. 2a).

This magnetic couple should act against a couple of frictional rotational forces *F*_*fr*_, arising from the contact of the capsule with the surrounding tissue. The modulus *F*_*fr*_ of each frictional force is given by:

3 $$F_{Fr} = \mu \frac{N}{2}$$

where *N* is the normal force exchanged by the overall capsule with the contact surface and *µ* is the friction coefficient between the capsule and the surface. The frictional forces give rise to an antagonistic frictional torque *T*_*f*_, which tends to oppose the magnetic torque. Each force *F*_*fr*_ is applied on the same centroid of the magnetic force to generate the following modulus *T*_*f*_ of the frictional torque:

4 $$T_{f} = F_{Fr} \frac{L}{2}$$

where *L* is the length of the capsule. The rotation *β* actually achieved by the capsule corresponds to a new equilibrium state, characterized by the balance between the magnetic and the frictional torques, according to the following equilibrium condition:

5 $$T_{m} \ge T_{f} = MVB\sin \left( {\alpha - \beta } \right) \ge \frac{1}{4}\mu N$$

Regarding the force equilibrium, the magnetic force *F* (N) on the magnetic capsule is represented by:

6 $$\vec{F}_{M} = \left[ {\begin{array}{*{20}c} {F_{M,x} } \\ {F_{M,y} } \\ {F_{M,z} } \\ \end{array} } \right] = V\vec{M} \cdot \nabla \vec{B} = V\left[ {\begin{array}{*{20}c} {M_{x} \frac{{\partial B_{x} }}{\partial x} + M_{y} \frac{{\partial B_{y} }}{\partial x} + M_{z} \frac{{\partial B_{z} }}{\partial x}} \\ {M_{x} \frac{{\partial B_{x} }}{\partial y} + M_{y} \frac{{\partial B_{y} }}{\partial y} + M_{z} \frac{{\partial B_{z} }}{\partial y}} \\ {M_{x} \frac{{\partial B_{x} }}{\partial z} + M_{y} \frac{{\partial B_{y} }}{\partial z} + M_{z} \frac{{\partial B_{z} }}{\partial z}} \\ \end{array} } \right].$$

The friction and gravity forces are considered in addition to the magnetic forces. They can be expressed as follows (see Fig. 2b):

7 $$\vec{F}_{w} = m \cdot \vec{g}$$

8 $$\vec{F}_{f} = \mu_{s} (\vec{F}_{w} - \vec{F}_{M,x} )$$

The equilibrium of the acting forces for allowing attraction and locomotion of the endoscopic capsule is expressed by the following simple equations:

9 $$\vec{F}_{M,z} \ge \vec{F}_{f}$$

10 $$\vec{F}_{M,x} \ge \vec{F}_{w}$$

The selected values of magnetic forces and torques need to be generated at least at an electromagnetic source-capsule distance of 70 mm, corresponding to the typical gap between the abdominal wall and capsule.

In summary, the proposed toroidal EMG has to be designed to produce suitable values of magnetic forces and torques for the navigation of the endoscopic capsule, whose results are reported in Section III. However, it is noteworthy that the generation of the forces is the bottleneck in the generation of torques. In fact, when the magnetic capsule is actuated at low speeds and accelerations, the capsule quickly changes orientation and aligns its dipole moment with the applied field due to reduced frictional and inertial torques. In these conditions, it can be assume that the capsule dipole moment is approximately aligned with the applied field at all times and the capsule orientation can be controlled by adjusting the direction of the magnetic field. Therefore, the proposed toroidal EMG was designed with the force requirements for navigation taken into consideration. However, this implies that the chosen currents will be able to generate suitable values of torque for orientation.

### EMG Design and Optimization

A 3D (nonlinear) FEM model created in COMSOL Multiphysics 4.3 (COMSOL Inc., Sweden) is proposed for the dimensioning and design of the electromagnetic system. In particular, a design strategy driven by the following main specifications was considered: (i) minimization of the misalignment *α*-*β* for any applied magnetic field [Eq. (5)], and (ii) maximization of the magnetic force for any applied field gradient [Eq. (6)].

The optimization problem was solved for determining the values of the geometrical parameters reported in Fig. 2 in order to satisfy the requirements and constraints of the specific application [Eqs. (1)–(10)].

**Fig. 3:**
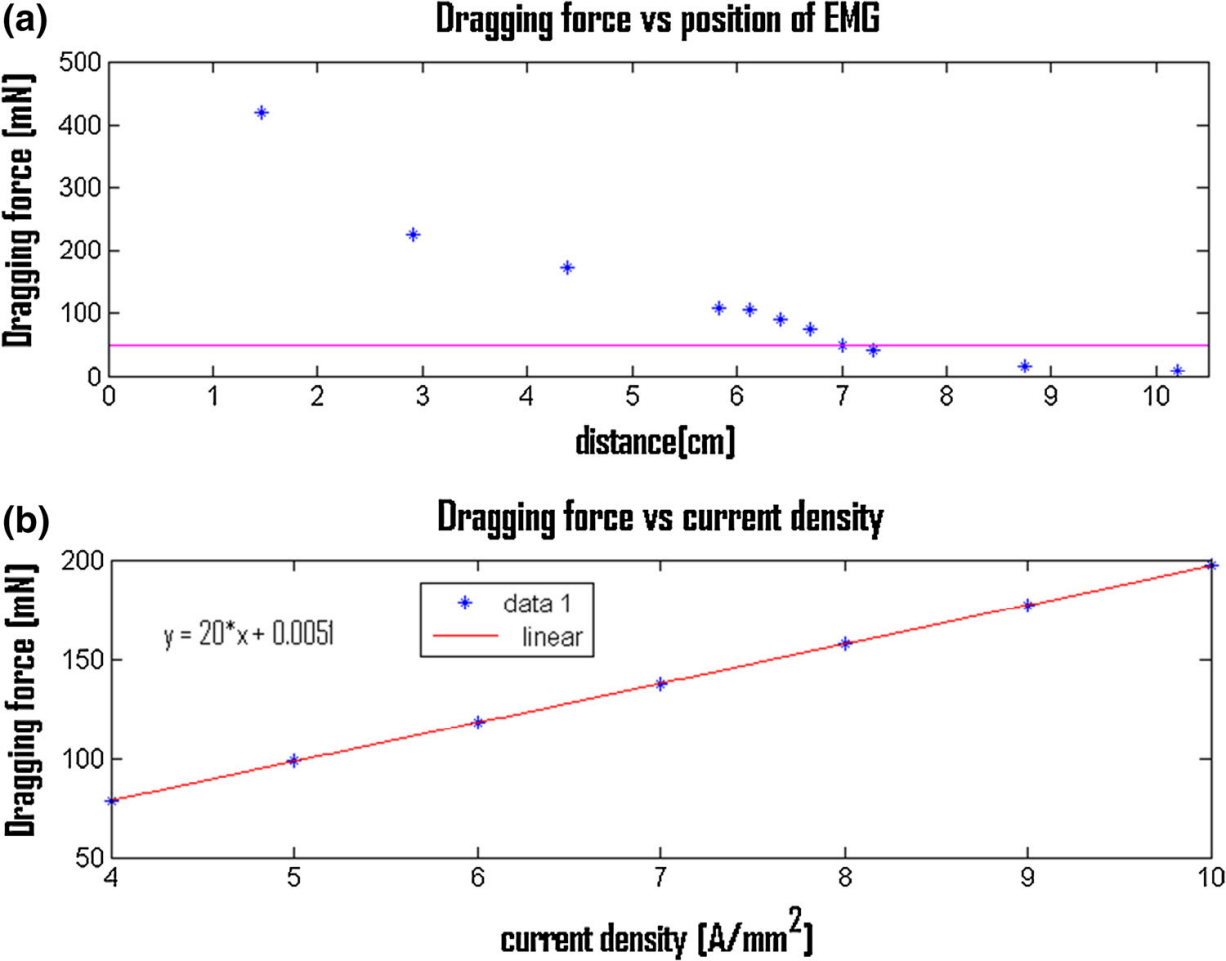
**a** Dragging force versus distance (measured from corner of EMG system along *x axis*, as represented in Fig. 2) between EMG system and capsule. Current density is set to 7 A/mm^2^; *magenta line* indicates minimum force needed for dragging endoscopic capsule (49.5 mN). **b** Dragging force versus current density inside EMG system. Distance is set to 5 cm (measured by a caliper). A linear trend is reported

From the FEM simulations, an electromagnetic control system was designed that is able to generate magnetic gradients of 0.175 T/m (82.5 mN) for attraction and 0.105 T/m for dragging (49.5 mN), and a magnetic field of 50 mT (5.3 mNmm) at a distance of 70 mm. These forces are capable of attraction and dragging of an endoscopic magnetic capsule in colonic tracts with a friction coefficient of no larger than 0.6 (as mentioned, the friction coefficient of the colon can range from 0.1 to 0.6 depending on the operation conditions, i.e., insufflation, cleaning, etc. [16]). Internal and external diameters of the EMG of 47 and 78 mm, respectively (copper wire with diameter of 3 mm and 5 windings), were selected in order to guarantee a high value of current density while restricting system overheating. Considering non-continuous operating conditions that allow current values larger than those usually recommended [20], a current density of 7 A/mm^2^ (63 A) was derived in the design phase by means of experimental experience. In addition, the EMG geometrical parameters *w*, *t*, *l*, *e*, and *α* were selected as 60 mm, 150 (EMG height of 93 mm), 150 mm (EMG height of 46 mm), 70 mm, and 150°, respectively, in consideration of the same experimental experience. Figure 3 shows the dragging force generated by the chosen EMG system with respect to its distance to the capsule and with respect to the supplied power (i.e., current). The results demonstrate that the chosen parameters are the best compromise for an endoscopic-capsule-based application (i.e., by respecting the distance between the endoscopic capsule and EMG system, encumbrance, etc.) in order to have (i) suitable values of torques and forces for the attraction and dragging of the endoscopic capsule, (ii) minimization of power consumption, and (iii) compatibility with the medical application (i.e., in terms of dimensions of the electromagnetic system with respect to the operating surrounding environment).

## Results

**Fig. 4:**
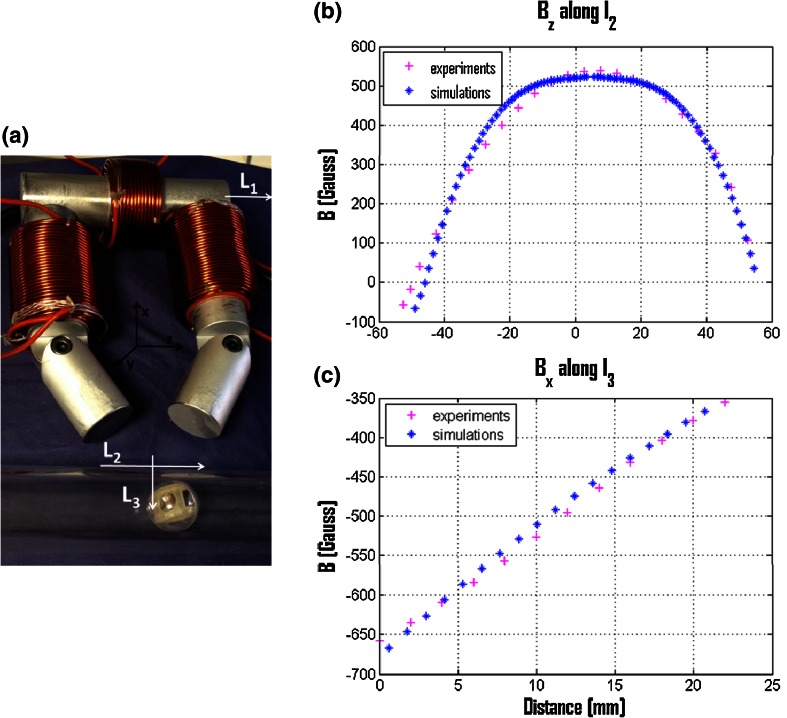
**a** The real system attracts the magnetic endoscopic capsule [14] into plastic tube. Lines *L1*, *L2*, and *L3* are directions along which experimental measurements of magnetic flux density components were performed. Comparisons between experimental measurements (*pink pluses*) and simulations of magnetic flux density (*blue stars*) for **b** measured and simulated *z* component along *L2* and **c** measured and simulated *x* component along *L3*

The modeling method was used to design and build the toroidal EMG (weight of 12.6 kg) used for the preliminary experimental evaluation (Fig. 4). In particular, tests were performed to (i) assess the design of the EMG (for confirming the FEM simulation results) and (ii) to validate the electromagnetic system control in an in vitro colon-like test bench, which consisted of a rigid polyurethane tube arranged to mimic the anatomical shape of the human colon, from the rectum to the cecum (about 900 mm in total length).

Simulation results were validated by comparing the magnetic flux density and the magnetic forces obtained by FEM simulations with experimental measurements, respectively. Firstly, tests consisted of powering the EMG with a current of 30 A. The produced magnetic flux density components were measured using a Hall-effect probe (Magnetometer KOSHAVA 5, Wuntronic, GmbH, Germany) supported and precisely moved by a robotic arm (RV-6SL, Mitsubishi Electric, Japan). In particular, the B_z_ component along lines L1 and L2 and the B_x_ component along line L3 (Fig. 4a) were considered. They correspond to the losses (L1) and to the locomotion (L2) and attraction (L3) components. The probe was moved in incremental steps of 2 mm (for L1 and L3) and 5 mm (L2) to reproduce the conditions used in the simulation scenario.

The obtained results show that the mean errors and standard deviations are 8.4 ± 0.63 Gauss, 20.5 ± 1.74 Gauss, and 0.4 ± 0.03 Gauss for L1, L2, and L3, respectively. Two examples of the obtained results for L2 and L3 are shown in Fig. 4b, c.

**Fig. 5:**
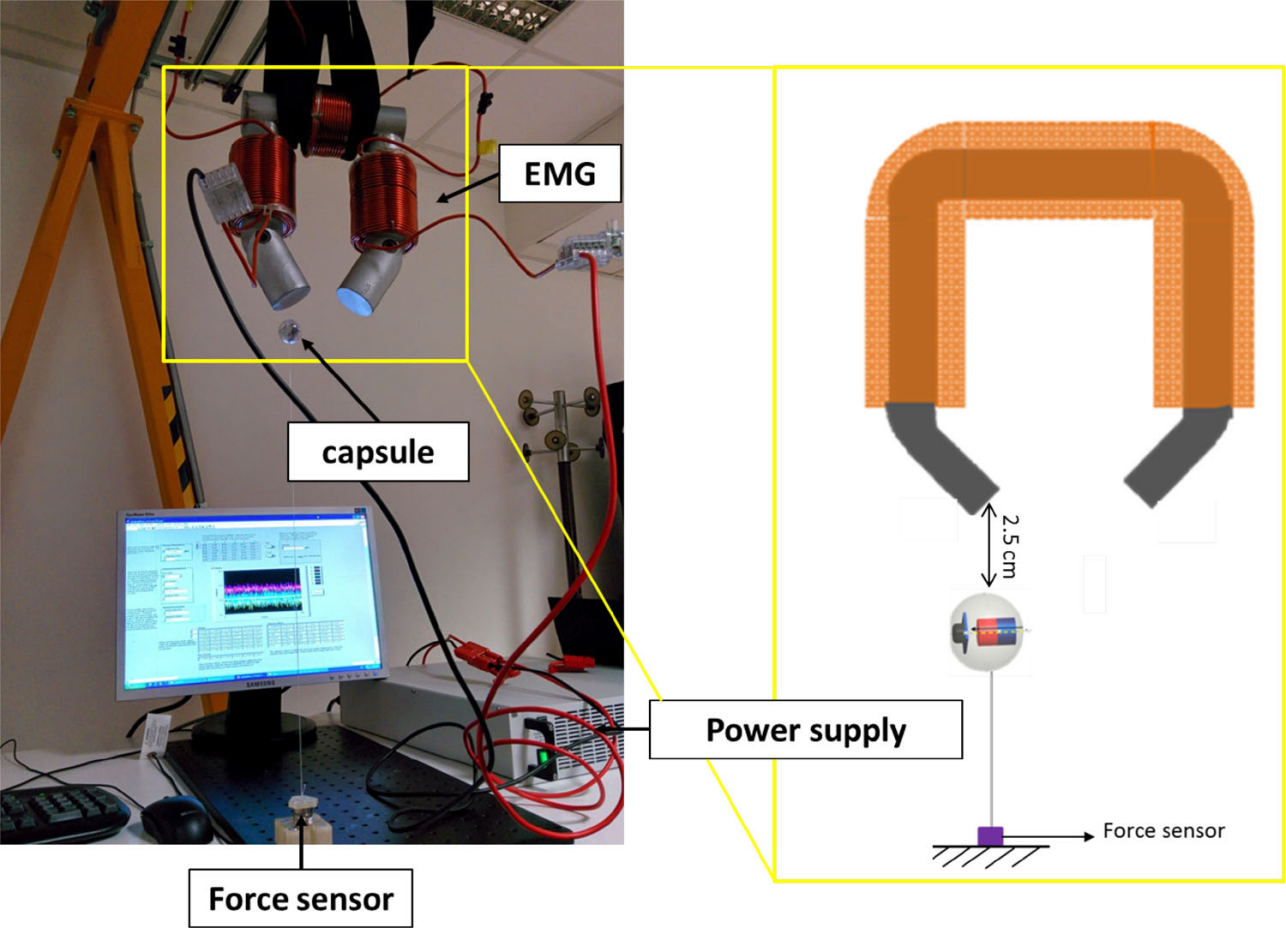
Force measurement set-up (photograph and sketch) for comparison between experimental and simulated magnetic forces. Capsule was constrained to force sensor by inextensible wire. Capsule was kept at 2.5 cm from corner of EMG corner with respect to *x axis*

**Fig. 6:**
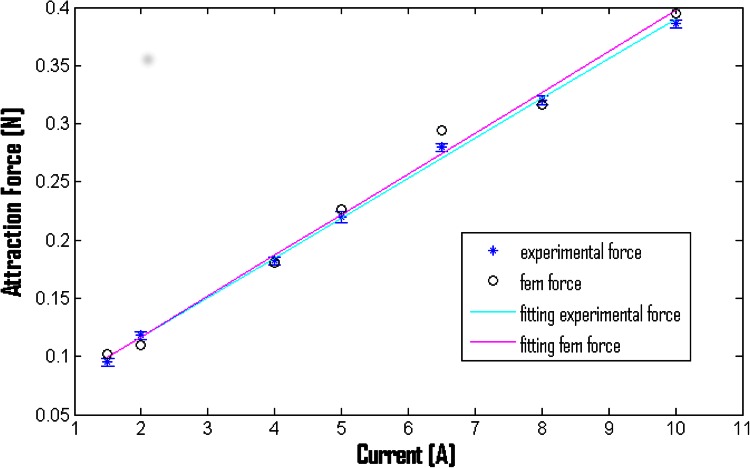
Comparison between experimental (*blue stars*) and simulated (*black circles*) magnetic forces. *Cyan* and *magenta* lines represent, respectively, linear trends obtained by fitting experimental and FEM data. Equations and R coefficients are respectively F_experimental_ = 0.03422I + 0.04783 (R = 0.9976) and F_fem_ = 0.03508I + 0.04659 (R = 0.9915)

In the second session of tests, a comparison between experimental and simulated magnetic attraction forces was made. The experimental magnetic forces were measured using a 6-axis force sensor (Nano17, ATI Industrial Automation, USA, resolution on Z axis of 1/320) attached to an endoscopic magnetic capsule (weight of 8.4 g and with an N52 NdFeB magnet of 511 mm^3^ in volume). The EMG system was supported by a block and tackle system due to the high weight and was placed at a distance of 2.5 cm from the capsule by an inextensible wire (Fig. 5). A current ranging from 1.5 to 10 A was imposed. For each value of current, 5 tests were repeated. The force sensor acquired data for 10 s and the mean value was calculated. Then, the current inside the electromagnet was increased. An image of the set-up and the results are given in Figs. 5 and 6, respectively, considering the mean value and the standard deviations for experimental forces and the magnetic force obtained by FEM simulations. The results show that both experimental and FEM magnetic forces have a linear trend with similar slopes (0.03422 versus 0.03508, difference of 0.86 mN/A). The obtained mean error is 7.1 ± 4.1 mN, corresponding to a percentage error of 3.67 ± 2.55 %. The results demonstrate that the simulated magnetic forces are a good approximation of the real magnetic forces.

Errors (below 10 % for both magnetic flux density and magnetic forces) could be due to constructive differences between the real system and the simulated model and larger losses by an inextensible wire. Future investigations will focus on matching the real system with the model; however, the present results demonstrate that the FEM simulations are a reliable tool for EMG design.

The second set of tests was performed to preliminary evaluate the navigation of a magnetic capsule. A scaled prototype of a capsule, already developed within a European project [21], was used as the capsule prototype. It consisted of the shell (external diameter of 37 mm and internal diameter of 33 mm), an internal mechanical frame with bearing balls, and an NdFeB N52 magnet (diameter of 11.4 mm and height 5.5 mm, compatible with capsule limitations). The total weight of the capsule was 18.5 g. Since the prototype was fabricated with off-the-shelf components and thus not optimized for the target application, the weight was larger than that supposed in the model (i.e., 8 g). Therefore, the electromagnet-capsule distance was reduced to reproduce the conditions in the real experiments. Based on the above considerations, an in vitro experimental evaluation was performed for a proof-of-concept study of the system.

The endoscopic capsule was controlled by the user by moving the external EMG at a distance of 30 mm. Navigation in a plastic tube, which mimics the human colonic tract, was performed. The qualitative results show the possibility of the navigation of the magnetic capsule by using the toroidal EMG (Fig. 4a and attached video).

## Discussion and Conclusion

This study demonstrated the feasibility of using a toroidal EMG with a ferromagnetic core for the optimal maneuvering of a magnetic endoscopic capsule. Compared to a previous work, in which the authors used a single EMG [21], the toroidal EMG has several advantages. Firstly, for a given number of windings, the radial size is reduced, thus maximizing the height (*t* + *t* + *l* parameters, as depicted in Fig. 2b); consequently the generated heat can be dissipated easier and faster. Secondly, the magnetic poles are closer to each other, allowing more reliable control of the capsule. The electronic safety for patients is presently guaranteed by insulation of the wires and connections and by the fact that the electromagnet is Class III equipment with a feeding DC voltage of 18 V. In the future, the system will be covered by an insulating jacket to improve the electronic safety for patients and the medical doctor.

The experimental evaluation was performed by using the designed electromagnetic source in order to (i) confirm the FEM simulation results, (ii) test the electromagnetic system, and (iii) evaluate the magnetic navigation principle in in vitro conditions mimicking the colonic tract. In particular, tests were performed with the developed electromagnetic source and with a capsule prototype in in vitro conditions in order to evaluate the magnetic navigation principle and test the electromagnetic system. Tests aimed to demonstrate the possibility of fine tuning and maneuvering of the electromagnetic system for effective navigation. Although the in vitro setup does not completely reproduce a real clinical environment and conditions, it was used in order to demonstrate the effective maneuverability of the capsule. In addition, the use of a rigid plastic tube guarantees higher repeatability and reduction of random effects in the tests, conditions needed at this level for an accurate validation of the model. Further investigations in ex vivo and in vivo conditions are needed to overcome the limitations of an in vitro setup in order to have a more realistic scenario and to assist the clinical transfer of the platform. However, specific parameters typical of an in vivo setting (e.g., friction of colon lumen, distances between electromagnetic system and capsule) obtained from the literature were considered in this study for the theoretical analysis and design of the electromagnetic navigation system.

In addition, future works will focus on the evaluation and compensation of a large number of possible sources of instability, which may render the capsule’s control more challenging in an in vivo application. The authors believe that the most appropriate solution could be a combination of magnetic field modulation based on a localization feedback [22, 23] and the motion of the external electromagnet assisted by a robotic manipulator. Finally, the need of a cooling system due to high power consumption will be evaluated with regards to real operating conditions.

## Electronic supplementary material

(TIF 5.01 Mb)
